# Apico-Aortic Conduit for TAVR Failure

**DOI:** 10.1016/j.jaccas.2025.105927

**Published:** 2025-11-03

**Authors:** Igor Belluschi, Giuseppe Bruschi, Bruno Merlanti, Benedetta De Chiara, Alessandro Costetti, Antioco Cappai, Fabrizio Settepani, Claudio Francesco Russo

**Affiliations:** ASST Grande Ospedale Metropolitano Niguarda, Heart Transplant & Cardiac Surgery Unit, “De Gasperis” Cardio-Thoracic and Vascular Department, Milan, Italy

**Keywords:** aortic hypoplasia, aortic stenosis, apico-aortic conduit, TAVR

## Abstract

**Background:**

The apico-aortic conduit (AAC) has been described as a valid alternative in patients with extreme aortic calcifications or patent grafts below the sternum.

**Case Summary:**

A 54-year-old woman presented with progressive dyspnea 10 years after transcatheter aortic valve replacement (TAVR). Her prior surgical history included triple coronary artery bypass graft with both internal mammary arteries. Owing to a critically narrowed residual aortic root area observed on computed tomography, a beating-heart AAC procedure was performed.

**Discussion:**

AAC has historically been proposed as a last-resort option in patients at prohibitive risk for conventional surgery. In our case, TAVR was first performed in a small aortic box. After the failure of this percutaneous approach, conventional surgical reintervention became prohibitive owing to massive aortic calcifications.

**Take-Home Messages:**

When both conventional surgery and TAVR are not feasible, AAC may represent a viable choice. Before any percutaneous approach, emergent or lifetime bailout surgical strategies should be carefully planned.

## Central Illustration

**Figure:**
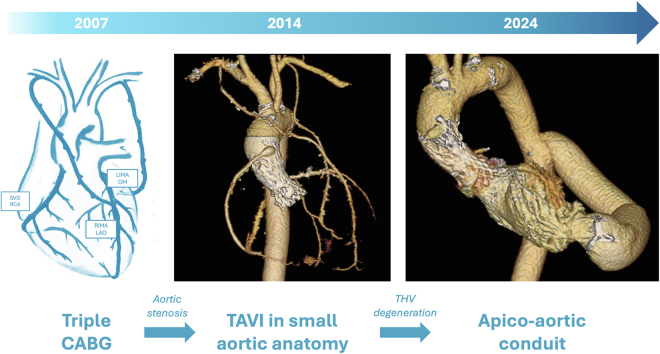

## History of Presentation

A 54-year-old woman with familial hypercholesterolemia presented with worsening dyspnea, resulting in advanced NYHA functional class III and angina. She underwent a transcatheter aortic valve replacement (TAVR) 10 years prior.

**Take-Home Messages**

• When both conventional surgery and TAVR are not feasible, AAC may represent a viable choice.
• Before any percutaneous approach, emergent or lifetime bailout surgical strategies should be carefully planned.

## Past Medical History

In 2007, at the young age of 37 years, the patient underwent a triple coronary artery bypass graft procedure with both internal mammary arteries (right on left anterior descending artery; left on marginal artery) and a saphenous vein graft on the right coronary artery, after a percutaneous right coronary ostium intervention 1 year before.

Seven years later, she developed severe symptomatic aortic stenosis in the context of a bicuspid aortic valve. Considering both graft patency, located just below the sternum, and the evidence of a porcelain and hypoplastic aorta, the Heart Team decided to perform a TAVR (Society of Thoracic Surgeons mortality score: 5.1%) with a 23-mm self-expandable (SE) CoreValve Evolut R prosthesis (Medtronic Inc). During the post-procedure stay, she required a permanent pacemaker implantation owing to complete atrioventricular block. After 4 days, she was asymptomatic and was discharged, despite transthoracic echocardiography (TTE) showing increased gradient (mean 25 mm Hg) in a background of very small aortic root.

**Visual Summary:**
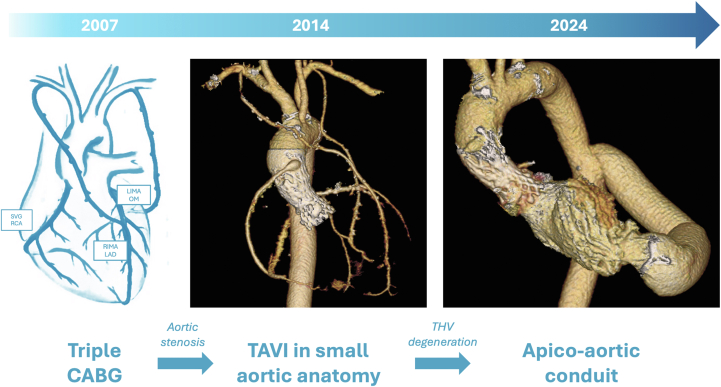
Procedure Timeline

## Investigations

**Figure 1:**
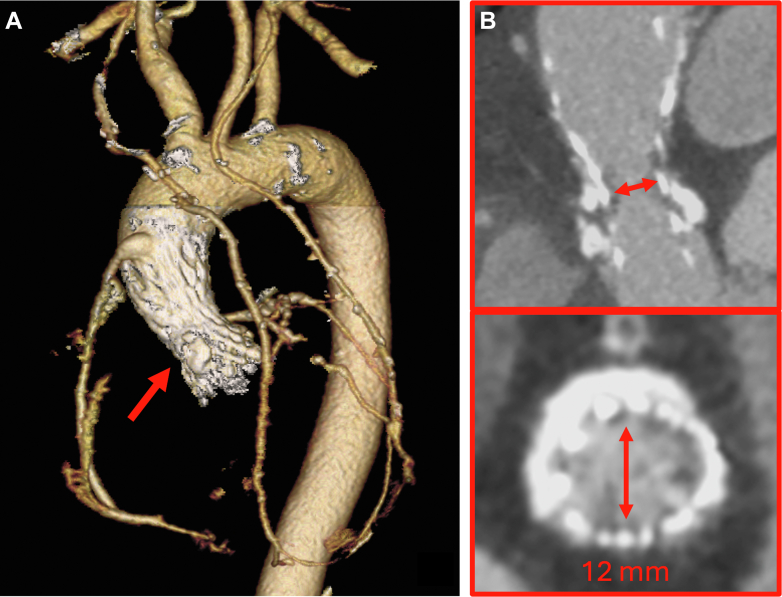
TAVR Failure in Calcified and Small Anatomy Baseline computed tomography showed coronary graft patency and an extremely small residual aortic root area after transcatheter aortic valve replacement (TAVR) (A), especially at the level of the sinotubular junction resulting in approximately 140 mm^2^ and a maximum diameter of 12 mm (red arrows) (B).

Follow-up TTE and recent transesophageal echocardiography revealed a progressive increase of transaortic gradients (mean from 25 mm Hg to 45 mm Hg, peak from 42 mm Hg to 87 mm Hg), mild paravalvular leak, moderate mitral regurgitation, and preserved left ventricular ejection fraction. Symptoms were initially trivial, but in the last year the patient experienced worsening functional class and angina requiring rehospitalization. On computed tomography, the smallest residual aortic area at the level of the sinotubular junction (STJ) was almost 140 mm^2^, with a maximum diameter of only 12 mm (Figure 1); however, both the left ventricle and the thoracic aorta appeared suitable for the anastomoses, without thrombi or massive calcifications at target sites.

## Management

**Figure 2:**
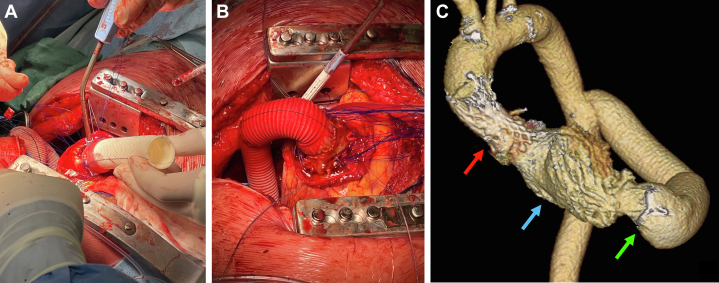
AAC Procedure Surgical beating-heart procedure. An apico-aortic conduit (AAC) including a biological valve prosthesis is sutured to the apex (A) first and then connected to the thoracic aortic graft (B). Post-procedure 3-dimensional computed tomography after AAC between the apex and the thoracic aorta. The red arrow points to the previous self-expandable prosthesis in the heavily calcified hypoplastic aortic root; the blue arrow points to contrast medium in the left ventricle; the green arrow shows the surgical bioprosthetic stent sutured at the beginning of the conduit to ensure 1-way flow and prevent regurgitation (C).

Given the severely limited residual area at the STJ, we opted for an apico-aortic conduit (AAC) beating-heart procedure (Video 1). Under femoral cardiopulmonary bypass, the subisthmic thoracic aorta was clamped down through a left anterolateral thoracotomy approach, and a terminolateral anastomosis with the thoracic aorta was performed using a 20-mm Gelweave vascular graft (Terumo Corp). During CO_2_ instillation and under ventricular fibrillation, the apex of the heart was exposed, incised by scalpel and partially resected by scissors. After ventriculotomy, the apex was joined to a custom-made 24-mm Cardioroot graft (Getinge) containing a 23-mm Inspiris Resilia biological prosthesis (Edwards Lifesciences, previously prepared on-bench by suturing the valve at the level of the graft pseudosinuses with a 2-0 polypropylene running suture. Twelve 3-0 single U-stitches with a pledget were applied to fix the sinusal portion of the graft to the ventricle. As a result, the biological valve was placed just below the apex. The 2 vascular grafts were finally connected (Figure 2) and covered by both vascular sealant (BioGlue; Cryolife) and dual-surface soft tissue membrane (Dualmesh; W. L. Gore & Associates, Inc). A de-airing maneuver was performed in a 2-step manner after both anastomoses of the grafts to the ventricle and aorta and then between each other.

## Outcome and Follow-Up

After uneventful stay, the patient was asymptomatic and was discharged in 3 weeks. Anticoagulation with warfarin was administered owing to both the occurrence of postoperative multiple asymptomatic atrial fibrillation episodes and the risk of thromboembolism in the valved conduit.

On predischarge, 6-month, and 12-month TTE, a stable residual mean transapical gradient of 12 mm Hg was identified. Both left ventricular function and sizes were preserved as well during follow-up, with a left ventricular ejection fraction of 60% and an end-diastolic diameter of 47 mm.

**Figure 3:**
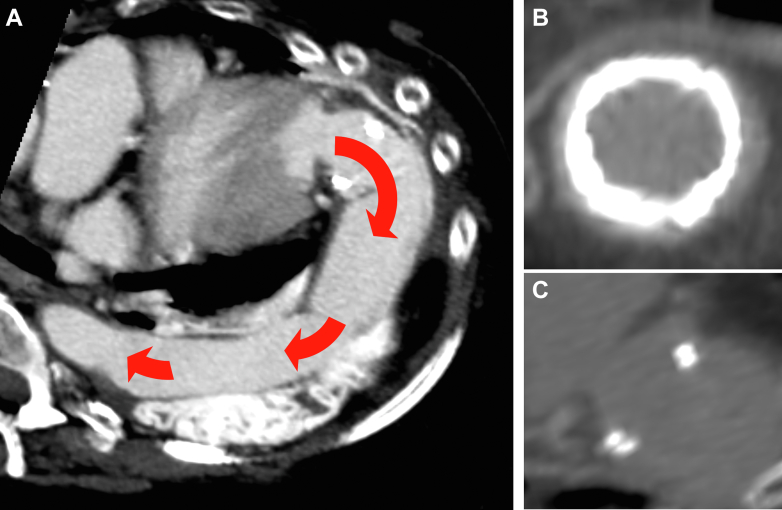
Follow-Up Imaging Follow-up computed tomography showed a good result and patency of the AAC with the absence of significant kinking (red arrows indicate blood flow) (A) and of subclinical conduit valve thrombosis (B and C).

A computed tomography scan performed 1 year later showed patency and no kinking of the AAC graft. In addition, no signs of conduit valve thrombosis, such as hypoattenuated leaflet thickening, were observed (Figure 3).

## Discussion

Percutaneous implantation of an aortic valve was performed 10 years ago as an off-label procedure owing to heavily calcified aorta and a moderate hypoplastic aortic box, resulting in only 16 to 18 mm STJ diameter. Indeed, even a surgical approach would have been challenging requiring strategies for annular enlargement (such as the Nicks, Konno, or Manougian procedures), but practically was not feasible owing to full aortic root calcifications.

To choose the best TAVR device, several transcatheter heart valve (THV) models have been evaluated for this procedure, but most of them were rejected, especially a balloon-expandable prosthesis because of the risk of annular rupture. Supra-annular SE prostheses have shown good hemodynamic results in the context of small aortic annuli.^1^ However, the smallest available size of an SE THV was a 23-mm prosthesis, which was too large for a tiny aortic root as in our case. Nevertheless, considering the patient's surgical risk as reported above, we clearly explained to her the limits of this approach: a prosthetic underexpansion resulting in elevated gradients—a likely occurrence—would have had a negative impact on the long-term hemodynamic performance of the device.

After obtaining the patient's agreement, the procedure was performed successfully despite the need for pacemaker implantation, as expected in a very small bicuspid anatomy. The postprocedural mean gradient was elevated, but the patient experienced significant symptomatic relief. After 10 years, along with THV degeneration, the gradient increased, and the patient became symptomatic.

Because of prior technical limitations that contraindicated surgery 10 years ago (porcelain aorta and graft patency), a conventional approach including resternotomy and standard surgical aortic valve replacement after calcified THV explant was judged unfeasible. Nevertheless, even a redo strategy, TAVR-in-TAVR, was rejected as the tiny residual aortic area represented a main contraindication.

Our Heart Team opted for an unconventional surgical solution: the creation of a biological heart conduit. Indeed, AAC has been proposed as an acceptable alternative in selected patients who are at extreme risk for standard surgical aortic valve replacement owing to porcelain aorta and high-risk resternotomies.^2^, ^3^, ^4^ To obtain the best hemodynamic performance, a 23-mm valve size was selected for ACC considering the patient's surface area and to allow feasibility of future conduit valve-in-valve procedures. Furthermore, despite, to our knowledge, having not yet been reported, it could be postulated that patients who underwent multiple redo TAVRs with narrowed residual area may also benefit from this technique as a final option in lifetime management. To our knowledge, this may be the first reported case of AAC used as a rescue strategy after underexpanded and degenerated TAVR in the context of a small and porcelain aorta.

## Conclusions

An AAC performed via a thoracotomy approach may represent a valid bailout solution for TAVR failure when TAVR-in-TAVR is not feasible.

## Funding Support and Author Disclosures

Dr Bruschi has been a consultant for Medtronic. All other authors have reported that they have no relationships relevant to the contents of this paper to disclose.

## Appendix

Video 1 Pre- and Post-Procedure Computed Tomography 3-Dimensional Reconstruction of the Case and How the Apico-Aortic Conduit Procedure was Surgically Performed
